# Auxin, the organizer of the hormonal/environmental signals for root hair growth

**DOI:** 10.3389/fpls.2013.00448

**Published:** 2013-11-12

**Authors:** Richard D.-W. Lee, Hyung-Taeg Cho

**Affiliations:** ^1^Department of Biological Sciences, Seoul National UniversitySeoul, Korea; ^2^Plant Genomics and Breeding Institute, Seoul National UniversitySeoul, Korea

**Keywords:** auxin, boron deficiency, ethylene, jasmonate, phosphate deficiency, root hair, root hair-specific genes, strigolactone

## Abstract

The root hair development is controlled by diverse factors such as fate-determining developmental cues, auxin-related environmental factors, and hormones. In particular, the soil environmental factors are important as they maximize their absorption by modulating root hair development. These environmental factors affect the root hair developmental process by making use of diverse hormones. These hormonal factors interact with each other to modulate root hair development in which auxin appears to form the most intensive networks with the pathways from environmental factors and hormones. Moreover, auxin action for root hair development is genetically located immediately upstream of the root hair-morphogenetic genes. These observations suggest that auxin plays as an organizing node for environmental/hormonal pathways to modulate root hair growth.

## INTRODUCTION

The root hair develops as a tubular structure from the root hair-forming root epidermal cell. The root hair development on the root epidermal cell consists of two major steps: the fate determination step, which produce hair or non-hair cells, and the root hair differentiation (or morphogenesis) step, where the root hair initiates and elongates from the root hair cell (Grierson and Schiefelbein, 2002, 2009). The fate determination step in *Arabidopsis* has been genetically well characterized. In the non-hair cell position, a complex of WEREWOLF (WER, a MYB transcription factor), GLABRA3/ENHANCER OF GLABRA3 [GL3/EGL3, basic helix-loop-helix (bHLH) transcription factors], and TRANSPARENT TEST GLABRA (TTG, a WD40 protein) positively modulates the expression of GLABRA2 (GL2, a homeodomain transcription factor); GL2, then, works as a negative regulator against root hair differentiation by inhibiting the expression of genes for root hair morphogenesis (Grierson and Schiefelbein, 2009). On the other hand, in the hair cell position, a Leu-rich repeat receptor-like protein kinase (LRR-RLK) called SCRAMBLED (SCM) is likely to receive external signals from the inner tissues and suppress the expression of WER, and thus of GL2, so as to release the inhibition against root hair morphogenesis of the root hair cell (Grierson and Schiefelbein, 2009). The lack of GL2 in the hair cell seems to lead to the activation of a bHLH transcription factor, ROOT HAIR DEFECTIVE 6 (RHD6), which is necessary for root hair initiation (Masucci and Schiefelbein, 1996; Menand et al., 2007). RHD6 then positively controls other downstream bHLH transcription factors (Yi et al., 2010) and root hair-specific (RHSs) morphogenetic genes, *RHS* (Won et al., 2009).

Partially, independent of the developmental genetic pathway, auxin-related environmental factors and phytohormones affect the hair morphogenetic process (Masucci and Schiefelbein, 1994, 1996; Okada and Shimura, 1994; Katsumi et al., 2000; Lee and Cho, 2008). Root hair-modulating phytohormones include auxin, ethylene, jasmonic acid (JA), brassinosteroid (BR), and strigolactone (SL). Amongst these phytohormones, auxin has been most intensively studied regarding its role in root hair growth. Auxin shows an obvious positive effect on root hair elongation without affecting the fate determination step (Masucci and Schiefelbein, 1994, 1996; Pitts et al., 1998; Cho and Cosgrove, 2002). Auxin genetically works downstream of RHD6 as exogenous auxin restores root hairs in the root hair-defective *rhd6* mutant (Masucci and Schiefelbein, 1996). Recent studies have added diverse hormonal and environmental factors affecting root hair development and shown that majority of these factors work together with auxin to control root hair development. In this review, we divide the upstream pathway of root hair development into fate-determining pathway and environmental/hormonal pathway and locate auxin at the organizing node where diverse environmental and hormonal signals for root hair growth converge.

## AUXIN SIGNALING AND HOMEOSTASIS OPERATE CELL-AUTONOMOUSLY FOR ROOT HAIR GROWTH

Among three major nuclear auxin signaling components, auxin receptors [TRANSPORT INHIBITOR RESPONSE1 [TIR1]/ AUXIN SIGNALING F-BOX PROTEINs (AFBs)] and their substrates or auxin-signaling repressors [AUXIN/INDOLE-3-ACETIC ACIDs (Aux/IAAs)] have been well defined to affect root hair growth. The *tir1* mutant, along with the mutants of its paralogs *afb1, afb2, *and* afb3*, showed a decreased root hair growth (Dharmasiri et al., 2005), whereas RHS over-expression of TIR1 considerably enhanced root hair growth (Ganguly et al., 2010). These results are in accordance with the auxin receptors’ nature in that they cause degradation of repressors (Aux/IAAs) for auxin responses. Conversely, the genetic data have demonstrated diverged roles of these repressors during root hair development. Degradation-resistant mutants of *AUXIN RESISTANT2* (*AXR2*)/*IAA7* (Wilson et al., 1990; Masucci and Schiefelbein, 1996), *AXR3*/*IAA17* (Leyser et al., 1996), *SOLITARY* *ROOT* (*SLR*)/*IAA14* (Fukaki et al., 2002), and *IAA28* (Rogg et al., 2001) showed inhibition of root hair growth, indicating their negative function in auxin-mediated root hair growth, whereas the similar gain-of-function mutant of *SHY2/IAA3* (Knox et al., 2003) showed enhanced root hair growth, suggesting its positive role in root hair growth. In contrast, the role of another major auxin signaling component, AUXIN RESPONSE FACTORs (ARFs), has scarcely been characterized in root hair growth.

Cell type-specific gene manipulation experiments have demonstrated that auxin signaling and homeostasis for root hair growth are operational in a hair-cell autonomous way, where changes of auxin levels and auxin signaling components in the root hair cell directly affect root hair growth (Cho et al., 2007a; Lee and Cho, 2008). When genes were root hair-specifically expressed using a RHS promoter (Cho and Cosgrove, 2002; Kim et al., 2006a), the dominant *axr2-1* mutant gene specifically suppressed root hair growth (Won et al., 2009) while, as mentioned above, TIR1 greatly enhanced hair growth (Ganguly et al., 2010). In a complementary manner, when the dominant *axr3-1* mutant gene was expressed specifically in the non-hair cells to cause defects in auxin signaling exclusively within the non-hair cells, it did not show any effect on root hair growth in the hair cell (Jones et al., 2008). These studies together suggest that the auxin signaling for root hair growth is operational in the root hair cell.

Although the auxin signaling for root hair growth is hair cell autonomous, auxin concentration seems to be higher in the non-hair cell than in the hair cell. The expression of AUX1 (an auxin influx carrier) was shown to localize specifically to the non-hair cell, whereas PIN2 (PIN-FORMED2, an auxin efflux carrier) was evenly expressed in both hair and non-hair cells, which would cause more auxin accumulation in the non-hair cell than in the hair cell (Jones et al., 2008). Another study showed a similar result where exogenous auxin (1-naphthalene acetic acid, NAA) induced a much higher response of DR5::GUS, the auxin responsive reporter, in the non-hair cell than in the hair cell (De Rybel et al., 2012). Because defects of auxin signaling in the non-hair cell did not influence hair growth in the hair cell, the high accumulation of auxin in the non-hair cell is thought to provide sustainable auxin concentrations for the root hair cells where the non-hair cell file works as an auxin pipeline to supply auxin from the root tip auxin maximum to the root hair differentiation zone (Jones et al., 2008). The short-haired *pin2* mutant phenotype also supports this hypothesis (Cho et al., 2007b). PIN2 is mainly expressed and asymmetrically localized at the upper side (toward the shoot) in the epidermis of root meristem and elongation zones (Luschnig et al., 1998; Müller et al., 1998). Therefore, the loss of PIN2 would cause defects in supplying auxin from the root tip to the hair-differentiation zone, which in turn results in suppression of root hair growth.

Studies with various auxin transporters have demonstrated that the auxin homeostasis of the root hair cell is critical for root hair growth. RHS expression of auxin efflux carriers such as PINs (PIN1-4, PIN7, and PIN8) and P-GlycoProteins (PGPs)/ATP-Binding Cassette transporter Bs (ABCBs), dramatically suppressed *Arabidopsis* root hair growth, and this root hair inhibition was suppressed by the auxin efflux carrier inhibitor, suggesting that auxin efflux carrier-mediated root hair inhibition occurs due to depletion of auxin in the root hair cell (Lee and Cho, 2006; Cho et al., 2007a,b; Ganguly et al., 2010). Indole-3-butyric acid (IBA), an auxin precursor, provides another point-of-view on the relationship between auxin concentration and root hair growth. IBA goes through peroxisomal modification to be converted into IAA in the root cap. Interestingly, the loss-of-function mutant of an IBA-transporter, PLEIOTROPIC DRUG RESISTANCE8 (PDR8) /PENETRATION3/ABCG36, an IBA-specific efflux carrier protein, was shown to increase root hair length: under the *pdr8* mutant, IBA accumulation was increased, leading to an increased concentration of IAA (Strader and Bartel, 2009). As IBA serves an auxin reservoir, proper IBA transport from the root tip region to the hair-differentiation zone can be important for root hair growth.

Impairment of auxin transport caused by metabolite also supports the idea that changes in auxin concentration affect root hair development. D’orenon, a C_18_-ketone (5*E*,7*E*)-6-methyl-8-(2,6,6-trimethylcyclohex-1-enyl)octa-5,7-dien-2-one, an early cleavage product of β-carotene, was shown to affect auxin homeostasis by increasing abundance of PIN2 in the epidermal cells, leading to a decrease in auxin levels in the root hair cell similarly to RHS PIN overexpression aforementioned (Schlicht et al., 2008).

## CROSS-TALK BETWEEN AUXIN AND OTHER HORMONES FOR ROOT HAIR GROWTH

Many phytohormones have been shown to control root hair development by cross-talking with auxin. To date, the influence of auxin and ethylene on root hair development has been most heavily studied. However, the influence of other phytohormones, such as SL, BR, and JA has been steadily inching their way into root hair development.

Although the functional relationship between ethylene and auxin for plant development can be both positive and counteractive depending on the tissue type, both hormones are in a positive relationship in regard to root hair growth (Cary et al., 1995; Tanimoto et al., 1995; Masucci and Schiefelbein, 1996; Muday et al., 2012). For example, the root hair defect of *rhd6* mutant was rescued both by auxin and by ACC (1-aminocyclopropane-1-carboxylic acid, the ethylene precursor; Masucci and Schiefelbein, 1994), the long-haired phenotype of the ethylene overproducing *eto1 *mutant was suppressed by the *aux1* mutation (Strader et al., 2010), and the *aux1 ein2* double mutant showed an additive root hair defect (Rahman et al., 2002). These results consistently demonstrate a positive relationship between auxin and ethylene. A transcriptome analysis further demonstrates that auxin and ethylene act on the common pathway for root hair development since almost 90% of the genes were commonly up-regulated by both auxin and ethylene (Bruex et al., 2012). However, the pathways that these two hormones take for root hair growth seem to be complicated.

According to the experimental data so far reported, auxin appears to work both upstream and downstream of ethylene. Auxin was able to restore root hair growth in the ethylene-insensitive mutant *ein2-1* (Rahman et al., 2002). Similarly, auxin-resistant mutants, *ibr5*, *tir1*, *axr1*, and *aux1*, were shown to suppress the long root hair phenotype of the ethylene-overproducing mutant *eto1* (Strader et al., 2010). In addition, ethylene enhances auxin biosynthesis in the root tip and stimulates basipetal auxin transport toward the root elongation zone (Stepanova et al., 2005; Růžička et al., 2007; Swarup et al., 2007). These results suggest that auxin may work downstream of ethylene for root hair growth. On the other hand, a competitive inhibitor of ethylene, 1-methylcyclopropene (1-MCP), inhibited auxin-induced restoration of root hair growth in *rhd6* (Cho and Cosgrove, 2002), and ethylene was shown to initiate the auxin-induced microtubule randomization which is necessary for root hair elongation (Takahashi et al., 2003). The root hair growth of the auxin-signaling defective *arf7 arf9* double mutant did not respond to auxin, but ACC greatly enhanced root hair growth in this mutant (Kapulnik et al., 2011b). These latter cases suggest that ethylene may be working downstream of auxin for root hair growth.

Strigolactone positively affects root hair development via ethylene and auxin. The treatment of synthetic SL, GR24, under the *max2* (defective in MORE AXILLARY GROWTH2, the SL signaling component) mutant did not enhance root hair growth, whereas such growth was evident under the *max3* or *max4* mutant (SL biosynthetic mutants), signifying that SL affects root hair growth via the MAX2-mediated SL signaling pathway (Kapulnik et al., 2011a). SL seems to work via ethylene to stimulate root hair growth: *max2* is sensitive to ACC, but *ein2* and *etr1* are insensitive to GR24 in root hair growth (Kapulnik et al., 2011b). SL directly influences ethylene production by increasing the transcription level of ACS2 (ACC Synthase 2; Kapulnik et al., 2011b), an enzyme necessary for ethylene biosynthesis.

Unlike the SL-ethylene case, SL and auxin interact with each other in multiple levels for root hair growth. First, sub-effective concentrations of auxin and SL together enhanced root hair growth more greatly than when they were applied individually, indicating their synergistic effect on root hair growth (Kapulnik et al., 2011b). Second, while auxin failed to enhance root hair growth in the *arf7 arf9* double mutant, root hair growth in this mutant was normally stimulated by SL, suggesting that SL works independently or downstream of auxin for root hair growth (Kapulnik et al., 2011b). As ethylene was shown to enhance root hair growth of the *arf7 arf9* double mutant and SL was shown to work through ethylene, the effect of SL on the *arf7 arf9* double mutant could take place through ethylene. Third, auxin works downstream of SL for root hair growth as exogenous auxin could restore the defective root hair growth of *max2* and *max4* mutants almost to the wild-type level (Mayzlish-Gati et al., 2012). In addition, although they are not specified to the root hair, the results that SL modulates auxin transport and auxin signaling by regulating the expression of PINs (Bennett et al., 2006) and TIR1 (Mayzlish-Gati et al., 2012) suggest that SL superimposes the auxin action.

Brassinosteroid has been shown to inhibit root hair growth. Application of epi-brassinolide (epiBL, a synthetic BR) significantly inhibited root hair growth of the *Arabidopsis* seedling root, and this was phenocopied by Aux/IAA overexpression (Kim et al., 2006b). The expression of root hair-related Aux/IAAs, such as AXR2/IAA7, AXR3/IAA17, and SLR/IAA14, was increased by epiBL and suppressed in the BR-insensitive *bri1* mutant, suggesting a possibility that BR may inhibit root hair growth by suppressing auxin signaling in the root hair (Kim et al., 2006b). This observation and interpretation is interesting. Although auxin also induces expression of Aux/IAAs, it also simultaneously causes the degradation of these repressors. However, while BR stimulates the expression of Aux/IAAs, it would not cause their degradation, resulting in accumulation of Aux/IAA repressors and suppression of auxin signaling. This can be a rare case of auxin-BR interactions, probably specific to root hair growth, because auxin and BR generally show synergistic effects in diverse developmental processes (Hardtke et al., 2007).

Jasmonic acid positively affects root hair growth where exogenous JA enhanced root hair growth in a dosage-dependent manner (Zhu et al., 2006). JA also affects root hair morphogenesis as it increases branched root hairs (Zhu et al., 2006). Although the JA signaling to the root hair development has not been well characterized, it can be cross-connected with auxin and ethylene signaling. The interconnectivity between auxin and JA signaling is shown as the auxin signaling mutants *axr1* was resistant to exogenous JA in the primary root inhibition assay (Tiryaki and Staswick, 2002), which was exemplified when the JA response mutant, *jar1-1*, was found to be an allele of the *AXR1* gene (Tiryaki and Staswick, 2002). In addition, JA was shown to promote auxin biosynthesis by up-regulating *YUCCA8* and *YUCCA9* (Hentrich et al., 2013). However, it has not been directly shown whether JA affects root hair development via auxin. On the other hand, the crosstalk between JA and ethylene for root hair growth has been shown. JA-induced root hair growth was blocked by AVG or Ag^+^, the inhibitors of ethylene biosynthesis and signaling, respectively, and in the ethylene-insensitive *etr1-3* mutant (Zhu et al., 2006), suggesting that ethylene signaling is required for JA-mediated root hair growth. Conversely, the treatment of JA biosynthesis inhibitors, ibuprofen and SHAM, suppressed ethylene-mediated root hair growth, implying that JA and ethylene mutually require each other for root hair growth (Zhu et al., 2006). The likely converging point of JA and ethylene signaling is EIN3/EIL1 (ETHYLENE INSENSITIVE3/EIN3-LIKE1). JAZ (JA ZIM-DOMAIN, a transcriptional repressor), which is degraded by JA, represses EIN3/EIL1 by physically interacting with them, and JA treatment relieves JAZ from EIN3/EIL1 leading to the expression of ethylene-responsive genes and the increase of root hair growth (Zhu et al., 2011).

## THE INTERACTION BETWEEN ENVIRONMENTAL FACTORS AND AUXIN FOR ROOT HAIR GROWTH

Root hair growth is also affected by environmental factors including phosphate (Pi), boron, and glucose. Among these, the implication of Pi in root hair development has been most intensively studied. Due to the immobile nature of Pi ion in the soil, plant roots frequently experience Pi deficiency, which stimulates root hair formation and elongation (Schmidt and Schikora, 2001). Pi deficiency at least partly modulates root hair development by affecting auxin signaling and transport. The *APSR1* (*ALTERED PHOSPHATE STARVATION RESPONSE1 *encoding a potential transcription factor) gene plays a negative role in root hair elongation during normal Pi conditions and is down-regulated under low Pi states leading to an enhanced root hair growth (González-Mendoza et al., 2013). The loss of APSR1 caused a clear decrease in PIN7 protein levels. Although the decrease of PIN7 expression in the root hair cell can restore auxin levels and thus growth of the root hair (Ganguly et al., 2010), whether ASPR1 directly modulates root hair growth via PIN7 remains unknown. However, this study supports the idea that auxin mediates Pi deficiency-induced root hair growth. In contrast, a different study indicates that Pi deficiency-induced root hair growth and formation may work downstream or independently of auxin signaling. Auxin insensitive signaling and transport mutants such as *axr1*, *axr2*, and *aux1,* show shorter and fewer root hair phenotypes, and Pi deficiency restored both growth and number of root hairs (Schmidt and Schikora, 2001).

Phosphate was also shown to affect root hair development via the SL pathway. The response to Pi starvation was reduced under the defects in SL biosynthesis and signaling. *max2* and *max4* mutants showed a decrease in expression of Pi starvation-induced (PSI) genes while GR24 was able to rescue the reduced Pi response under the *max4-1* mutant (Mayzlish-Gati et al., 2012). Since SL was known to work upstream of ethylene (Kapulnik et al., 2011b), Pi may affect the ethylene signaling via SL, creating a linear signaling pathway from an external influence, in this case Pi, to the root hair development via phytohormones (Mayzlish-Gati et al., 2012). Complementing the ideas above, the *hsp2* (*HYPERSENSITIVE TO PHOSPHATE STARVATION 2*, an allele of *CTR1* or *CONSTITUTIVE TRIPLE RESPONSE 1*) mutant showed a hypersensitivity to Pi starvation, indicating that ethylene signaling is involved in Pi-mediated root hair development (Lei et al., 2011). In a similar fashion, the *etr1-*1 and *ein2-5*, ethylene signaling mutants, reduced the expression of *PT2* (a high-affinity phosphate transporter gene) while the ethylene over-producing *eto1-1* mutant increased *PT2* expression, further exemplifying the relationship between Pi and ethylene to guarantee better acquisition of Pi for the plant (Lei et al., 2011). However, because Pi deficiency could restore root hair growth and formation in the ethylene signaling mutants, *etr1* and *ein2*, Pi deficiency may also take an ethylene-bypassing pathway for root hair development (Schmidt and Schikora, 2001).

In addition to its effect on hair growth, Pi deficiency can affect the fate determination step in root hair development. Under Pi-deficient conditions, root hairs not only grow longer in the hair cells but also are formed ectopically in the non-hair cell position (Schmidt and Schikora, 2001; Müller and Schmidt, 2004). Pi deficiency greatly enhanced the root hair number in the non-hair cell position, and this increase occurred partly even in the *wer, gl2,* and *ttg* fate determination mutants (Müller and Schmidt, 2004). Recent finding of bHLH32, a negative regulator of PSI genes, provides a link between Pi deficiency and the fate determination pathway, where bHLH32 was shown to interact with TTG and GL3 and high Pi conditions did not inhibit root hair development under the* bhlh32 *mutant background (Chen et al., 2007)*.* However, it has to be elucidated how bHLH32, interacting with TTG and GL3, affects hair/non-hair cell fate determination.

Boron also is implicated in root hair development. Boron deficiency causes enhanced root hair growth and formation where at least ethylene signaling has been shown to be implicated. Low boron-mediated increase of root hair growth was shown to be blocked in the *ein2-1* mutant, and the ethylene responsiveness was considerably enhanced by low boron in the elongation and differentiation zone of the root (Martín-Rejano et al., 2011). Although it has not been directly shown whether boron deficiency-enhanced root hair development requires auxin, the possibility exist as low boron increased auxin-sensitive DR5:GUS reporter gene expression in the root and low boron-mediated inhibition of the primary root was suppressed in the *aux1* mutant, suggesting that low boron signaling may use auxin signaling (Martín-Rejano et al., 2011).

Besides environmental factors, artificial high glucose conditions affect root hair development, and this seems to be linked with the expression of auxin-related genes (Mishra et al., 2009). As auxin and glucose cause numerous common responses, Mishra et al. (2009) compared auxin- and glucose-responsive transcriptomes and analyzed the relationship between auxin and glucose on root hair development. High glucose up-regulated *YUCCA2*, *PIN1*, *PIN2*, *ARF*, and *ABP1* genes while down-regulated *TIR1* and several *SAUR*, *Aux/IAA, *and* GH3* genes in the whole seedling level. Oddly, glucose suppressed auxin-induced DR5:GUS reporter expression in the root. Although glucose effects on auxin biosynthesis and signaling are complicated in the whole seedling level, glucose seems to require auxin signaling for root hair growth because mutants such as *tir1, slr1, axr3, *and* axr2* showed defects in glucose-induced root hair growth (Mishra et al., 2009).

## THE POSITION OF AUXIN IN THE OVERALL SIGNALING FOR ROOT HAIR DEVELOPMENT

As mentioned earlier, auxin works downstream of RHD6 for root hair growth. Recently, a bHLH transcription factor, called RHD6-LIKE4 (RSL4), was found to be a direct downstream target of RHD6 (Yi et al., 2010). RSL4 is expressed in the hair cell file of the elongation and differentiation zone of the root, and its loss of function mutant *rsl4-1* grew much shorter and fewer root hairs than wild type, indicating that RSL4 is indeed needed for root hair growth and initiation. Consistently, the RSL4 overexpression (under the CaMV 35S promoter) lines kept growing root hairs more than four times longer than those of wild type. Auxin was able to increase transcription of *RSL4* not only in wild type but also in the *rhd6* mutant background, suggesting that *RSL4* is the target of auxin in the downstream of RHD6. Furthermore, auxin failed to restore root hair growth in the *rsl4-1* mutant background, which is contrasted to the auxin effect in the *rhd6* mutant. These data collectively showed that the auxin pathway and the fate determination pathway via RHD6 converge on to RSL4 to modulate root hair growth.

Root hair growth and morphogenesis should require root hair-specifically functioning genes, as well as essential house-keeping morphogenetic genes, in which the RHS genes should specify all the hair cell-specific events for root hair morphogenesis. Diverse *RHS* genes have been functionally identified and these *RHS* genes commonly carry the characteristic root hair-specific *cis*-element (RHE) on their promoters (Kim et al., 2006a; Won et al., 2009). The function of RHE has been conserved at least in the angiosperm lineage since RHE was shown to be cross-functional between monocots and dicots, suggesting that the RHE-binding or *RHS*-modulating transcription factor also has been conserved in angiosperms (Kim et al., 2006a). The expression of *EXPANSIN A7*, a *RHS* gene, has been demonstrated to be regulated by RHD6 (Cho and Cosgrove, 2002; Won et al., 2009) but in an indirect way (Yi et al., 2010). This leads us to a hypothesis that *RHS* genes are located downstream of RSL4. The transcriptome analysis with wild type, *rsl4-1* mutant, and *RSL4* overexpressor revealed that RSL4 indeed up-regulated many *RHS* genes (Yi et al., 2010). Moreover, Pi deficiency was able to restore root hair from *rhd6* but failed to do that in *rsl4-1*, suggesting that the Pi deficiency signaling via auxin also requires RSL4 to promote root hair growth (Yi et al., 2010). The comparison between three independent transcriptome analyses further indicates that auxin and RSL4 commonly act on *RHS* genes. Auxin up-regulated 97 genes in the *rhd6* mutant background (Bruex et al., 2012), and RSL4 overexpression up-regulated 83 genes (Yi et al., 2010). Won et al. (2009) found that 24 *RHS* genes were down-regulated in the *rhd6* mutant background, among which 16 *RHS* genes were found in both transcriptome collections from Bruex et al. (2012) and Yi et al. (2010). These results further suggest that auxin operates upstream of RSL4 to stimulate *RHS* gene expression.

Recently, a membrane-anchored MYB (maMYB), an R2R3-type MYB transcription factor, has been implicated in root hair growth (Slabaugh et al., 2011). The silencing of maMYB via RNAi shortened root hair length without affecting hair initiation, indicating that maMYB is specifically involved in hair elongation. The interesting point is that exogenous auxin rescued the short root hair phenotype of the maMYB–RNAi line and promoted the transcription of maMYB of the wild type plant. These results suggest that maMYB works upstream of RSL4 for root hair growth. The same study showed that maMYB affects the expression of a *RHS* gene (*RHS14*) but in a negative way. It is unlikely that maMYB directly binds RHE to suppress *RHS* genes because RHE works in a positive manner (Won et al., 2009). maMYB might modulate *RHS* genes by binding *cis*-elements other than RHE or by interacting some upstream factors to negatively regulate *RHS* genes. Not all *RHS* gene products seems to positively work for root hair growth. Some of them, such as RHS1 and RHS10, negatively regulate root hair growth (Won et al., 2009), suggesting that the overall root growth process is in a balance of both positive and negative modulators. maMYB may provide another level of regulatory tool to fine-tune root hair growth between RSL4 and auxin.

## CONCLUDING REMARKS

**FIGURE 1:**
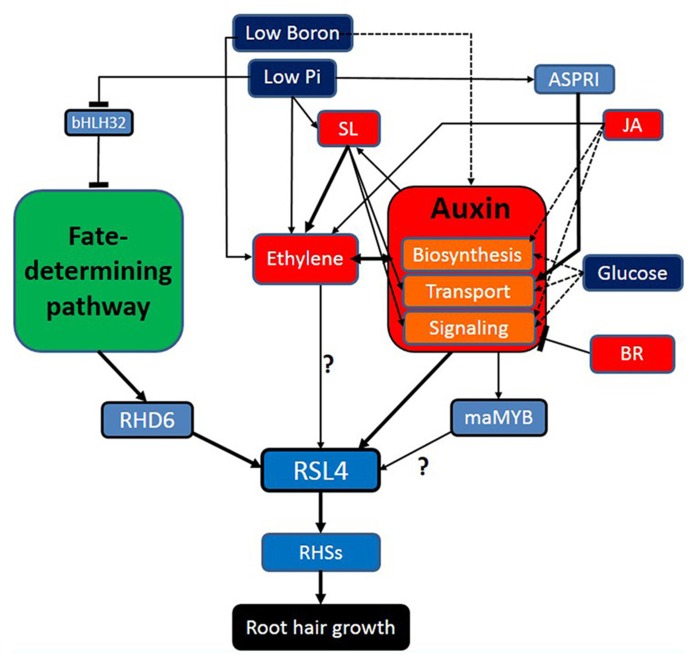
**Auxin plays an organizing center for environmental/ hormonal pathways for root hair growth.** Navy blue, red, light blue boxes indicate environmental factors, hormones, and genetic factors, respectively. Broken-lined arrows represent the cases where the direct effect on the root hair by the factor has not been shown. The blunt bar end indicates an inhibitory effect.

Root hair-controlling factors listed in this review can be classified largely into fate-determining developmental factors, hormonal factors, auxin-related environmental factors, and finally root hair morphogenetic genes (**Figure 1**). These factors show diverse interactions; not only linear but also networking and mutual. Environmental factors generally take advantage of hormonal signaling to modulate root hair growth, in which sometimes multiple hormones are implicated to mediate the environmental factor (e.g., Pi deficiency). Environmental factors maximize their effects on root hair development also by affecting the fate-determining developmental steps, resulting in increased root hair number. It is noticeable that most root hair-affecting hormones intensively interact with auxin in various levels; biosynthesis, transport, and signaling of auxin. In the overall signaling pathway for root hair growth, auxin funnels the upstream environmental pathways, and other hormonal signaling right onto the master regulator (RSL4) for root hair growth and morphogenesis. Considering these aspects, auxin is likely to play a role as an organizing center for environmental/hormonal signaling for root hair growth.

## Conflict of Interest Statement

The authors declare that the research was conducted in the absence of any commercial or financial relationships that could be construed as a potential conflict of interest.
